# Supplementary material for: Altered microRNA Transcriptome in Cultured Human Liver Cells upon Infection with Ebola Virus
Source: Int J Mol Sci. 2021 Apr 6;22(7):3792. doi: 10.3390/ijms22073792 (PMC8038836; doi:10.3390/ijms22073792)
Supplement: Supplementary file 1 [file ijms-22-03792-s001.zip › Supplementary_File/C_ GO_Analysis_Results/16-30nt_go_Reston-96h-Huh7_vs_Control-96h-Huh7_up.mature_mirna_targets/CC_result(Human).html]

| GO.ID | Term | Ontology | Count | Pop.Hits | List.Total | Pop.Total | Fold.Enrichment | Pvalue | FDR | Enrichment.Score | Gene.Ratio | GENES |
| --- | --- | --- | --- | --- | --- | --- | --- | --- | --- | --- | --- | --- |
| GO:0005622 | intracellular | Cellular component | 94 | 14673 | 103 | 18698 | 1.16296559495381 | 0.000457123701042206 | 0.302881790752346 | 3.33996626073868 | 0.912621359223301 | F5//ARFIP1//PI4KB//NDFIP1//GAL3ST1//BMI1//PSMD13//MIS18A//DYNC1I1//NFATC2//SYN1//GINS4//ZFP36L1//YY1AP1//SKIL//BRI3BP//GLS2//XAF1//MAPK3//TFAP4//AGMAT//ARL4C//CDO1//CFTR//CHN1//PLEKHH2//HECTD2//WIPF2//DCX//DUSP9//SEPHS2//CLIP3//LTN1//NIP7//TMEM214//MKS1//MAP2K7//SMAP1//DENND2D//CASP3//DPH6//REPS2//RASAL2//ZNF267//DMRT2//CREBL2//DUSP18//ZNF425//MIER3//DLX3//GRIK5//DND1//ACPP//CAMK1D//RORC//ZSCAN31//TEAD3//ZMAT4//EIF1AD//NFKBID//PRPF18//ZNF697//ZNF845//TNFRSF8//NFE2L3//DDIT4L//FAP//GLRB//GMPPA//PLS1//ARL8B//CYP26B1//PLXDC1//SCYL3//AIF1L//ABLIM2//ADAT2//SLC14A1//NOL4//SLC25A53//LRRTM1//ELOVL6//ZDHHC11//FMO1//TRAM2//BMP2//HS3ST1//CLEC4C//RTKN2//MYOT//MSR1//PLEKHB2//KCNH8//SHC4// |
| GO:0030864 | cortical actin cytoskeleton | Cellular component | 4 | 75 | 103 | 18698 | 9.68181229773463 | 0.000781935634718146 | 0.302881790752346 | 3.10682899455806 | 0.0388349514563107 | RTKN2//WIPF2//PLS1//PLEKHH2// |
| GO:0044424 | intracellular part | Cellular component | 92 | 14395 | 103 | 18698 | 1.16020327986052 | 0.00105779437981029 | 0.302881790752346 | 2.97559874477011 | 0.893203883495146 | BMI1//PSMD13//ARL4C//CFTR//CHN1//DDIT4L//PLEKHH2//HECTD2//DUSP18//ZNF425//DYNC1I1//DUSP9//FAP//SEPHS2//ARFIP1//GLRB//GMPPA//DND1//NFATC2//PI4KB//PLS1//MKS1//ARL8B//YY1AP1//MAPK3//MAP2K7//CYP26B1//CAMK1D//PLXDC1//SCYL3//SMAP1//ZFP36L1//AIF1L//CASP3//GINS4//ABLIM2//TNFRSF8//RASAL2//NFE2L3//TEAD3//ADAT2//SLC25A53//BRI3BP//BMP2//SLC14A1//GLS2//XAF1//TFAP4//AGMAT//CDO1//WIPF2//DCX//CLIP3//LTN1//NIP7//MIS18A//TMEM214//SYN1//DENND2D//DPH6//REPS2//GRIK5//LRRTM1//ELOVL6//ZDHHC11//NDFIP1//F5//FMO1//HS3ST1//RORC//TRAM2//ZNF267//DMRT2//CREBL2//MIER3//DLX3//ACPP//ZSCAN31//ZMAT4//EIF1AD//NFKBID//PRPF18//ZNF697//ZNF845//NOL4//MYOT//SKIL//RTKN2//GAL3ST1//CLEC4C//MSR1//PLEKHB2// |
| GO:0030863 | cortical cytoskeleton | Cellular component | 4 | 105 | 103 | 18698 | 6.91558021266759 | 0.00271167039840596 | 0.58233121805768 | 2.56676309978016 | 0.0388349514563107 | PLEKHH2//PLS1//RTKN2//WIPF2// |
| GO:0055038 | recycling endosome membrane | Cellular component | 3 | 72 | 103 | 18698 | 7.56391585760518 | 0.00735316954313806 | 0.89146677501205 | 2.13352542031951 | 0.029126213592233 | CFTR//CLIP3//PLEKHB2// |
| GO:0044464 | cell part | Cellular component | 100 | 16886 | 103 | 18698 | 1.07505614463179 | 0.00790086266554226 | 0.89146677501205 | 2.10232548713606 | 0.970873786407767 | ARL4C//CFTR//CHRNA1//PLEKHH2//KCNH8//WIPF2//CLEC4C//F5//FAP//CLIP3//GLRB//GRIK5//SHC4//MSR1//TNFRSF11B//ACPP//MAPK3//PLXDC1//SMAP1//SLC14A1//VIPR2//CASP3//SYT16//SLC22A6//TNFRSF8//CAMK1D//BMP2//ARL8B//SYN1//MYOT//LRRTM1//RTKN2//BMI1//PSMD13//CHN1//DDIT4L//HECTD2//DUSP18//ZNF425//DYNC1I1//DUSP9//SEPHS2//ARFIP1//GMPPA//DND1//NFATC2//PI4KB//PLS1//MKS1//YY1AP1//MAP2K7//CYP26B1//SCYL3//ZFP36L1//AIF1L//GINS4//ABLIM2//RASAL2//NFE2L3//TEAD3//ADAT2//IGLL5//SKIL//DCX//NDFIP1//RORC//SLC25A53//GAL3ST1//BRI3BP//GLS2//XAF1//TFAP4//AGMAT//LTN1//ZNF267//DMRT2//CREBL2//MIER3//DLX3//NIP7//MIS18A//ZSCAN31//ZMAT4//EIF1AD//NFKBID//PRPF18//DPH6//ZNF697//ZNF845//NOL4//TMEM214//ELOVL6//ZDHHC11//CDO1//DENND2D//REPS2//FMO1//HS3ST1//TRAM2//PLEKHB2// |
| GO:0043229 | intracellular organelle | Cellular component | 81 | 12604 | 103 | 18698 | 1.16663380095085 | 0.00814442669708195 | 0.89146677501205 | 2.08913948115986 | 0.786407766990291 | SMAP1//BMP2//SLC14A1//BRI3BP//GLS2//XAF1//MAPK3//TFAP4//AGMAT//ARL4C//ZNF267//DMRT2//CREBL2//DUSP18//ZNF425//MIER3//DLX3//DYNC1I1//DUSP9//GRIK5//DND1//NFATC2//NIP7//MIS18A//ACPP//YY1AP1//MAP2K7//CAMK1D//PSMD13//RORC//ZSCAN31//BMI1//ZFP36L1//TEAD3//ZMAT4//CASP3//EIF1AD//NFKBID//PRPF18//DPH6//ZNF697//ZNF845//TNFRSF8//NFE2L3//LRRTM1//TMEM214//ELOVL6//ZDHHC11//SCYL3//SYN1//NDFIP1//LTN1//NOL4//MKS1//WIPF2//DCX//TRAM2//AIF1L//ABLIM2//MYOT//PLEKHH2//PI4KB//CFTR//F5//SKIL//PLS1//ARL8B//CLIP3//CLEC4C//MSR1//RTKN2//PLEKHB2//ARFIP1//GAL3ST1//GINS4//ADAT2//DENND2D//SLC25A53//FMO1//CYP26B1//HS3ST1// |
| GO:0005623 | cell | Cellular component | 100 | 16915 | 103 | 18698 | 1.07321300965134 | 0.00898825645354803 | 0.89146677501205 | 2.04632454475626 | 0.970873786407767 | ARL4C//CFTR//CHRNA1//PLEKHH2//KCNH8//WIPF2//CLEC4C//F5//FAP//CLIP3//GLRB//GRIK5//SHC4//MSR1//TNFRSF11B//ACPP//MAPK3//PLXDC1//SMAP1//SLC14A1//VIPR2//CASP3//SYT16//SLC22A6//TNFRSF8//ARFIP1//PI4KB//NDFIP1//GAL3ST1//BMI1//CAMK1D//PSMD13//MIS18A//DYNC1I1//NFATC2//SYN1//GINS4//ZFP36L1//YY1AP1//SKIL//BRI3BP//GLS2//XAF1//TFAP4//AGMAT//CDO1//CHN1//HECTD2//DCX//DUSP9//SEPHS2//LTN1//NIP7//TMEM214//MKS1//MAP2K7//DENND2D//DPH6//REPS2//RASAL2//ZNF267//DMRT2//CREBL2//DUSP18//ZNF425//MIER3//DLX3//DND1//RORC//ZSCAN31//TEAD3//ZMAT4//EIF1AD//NFKBID//PRPF18//ZNF697//ZNF845//NFE2L3//DDIT4L//GMPPA//PLS1//ARL8B//CYP26B1//SCYL3//AIF1L//ABLIM2//ADAT2//NOL4//SLC25A53//LRRTM1//ELOVL6//ZDHHC11//FMO1//TRAM2//BMP2//HS3ST1//RTKN2//IGLL5//MYOT//PLEKHB2// |
| GO:0045211 | postsynaptic membrane | Cellular component | 5 | 244 | 103 | 18698 | 3.71995861849435 | 0.0112972728574592 | 0.89146677501205 | 1.94702638181741 | 0.0485436893203883 | GRIK5//CHRNA1//GLRB//LRRTM1//SHC4// |
| GO:0030867 | rough endoplasmic reticulum membrane | Cellular component | 2 | 31 | 103 | 18698 | 11.7118697150016 | 0.0125935194957359 | 0.89146677501205 | 1.89985288117303 | 0.0194174757281553 | CFTR//PI4KB// |
| GO:0055037 | recycling endosome | Cellular component | 4 | 165 | 103 | 18698 | 4.40082377169756 | 0.0131550149256751 | 0.89146677501205 | 1.88090865482764 | 0.0388349514563107 | CFTR//CLIP3//PLEKHB2//DYNC1I1// |
| GO:0044448 | cell cortex part | Cellular component | 4 | 166 | 103 | 18698 | 4.37431278512107 | 0.0134240929812917 | 0.89146677501205 | 1.8721150483865 | 0.0388349514563107 | RTKN2//PLEKHH2//WIPF2//PLS1// |
| GO:0005791 | rough endoplasmic reticulum | Cellular component | 3 | 90 | 103 | 18698 | 6.05113268608414 | 0.013491348166655 | 0.89146677501205 | 1.8699446498771 | 0.029126213592233 | PI4KB//CFTR//TRAM2// |
| GO:0015629 | actin cytoskeleton | Cellular component | 7 | 487 | 103 | 18698 | 2.60931799605271 | 0.0181168129217904 | 1 | 1.74191820026956 | 0.0679611650485437 | WIPF2//PLS1//AIF1L//PLEKHH2//RTKN2//ABLIM2//MYOT// |
| GO:0005884 | actin filament | Cellular component | 3 | 101 | 103 | 18698 | 5.39209843314429 | 0.0183280651272172 | 1 | 1.73688338058749 | 0.029126213592233 | WIPF2//PLS1//AIF1L// |
| GO:0005737 | cytoplasm | Cellular component | 74 | 11544 | 103 | 18698 | 1.16367936270849 | 0.0204177853990378 | 1 | 1.68999136514507 | 0.718446601941748 | NDFIP1//F5//ARFIP1//PI4KB//GAL3ST1//ZFP36L1//SKIL//BRI3BP//GLS2//XAF1//MAPK3//TFAP4//AGMAT//ARL4C//CDO1//CFTR//CHN1//PLEKHH2//HECTD2//WIPF2//DCX//DYNC1I1//DUSP9//SEPHS2//CLIP3//LTN1//NFATC2//NIP7//MIS18A//TMEM214//MKS1//MAP2K7//PSMD13//SMAP1//BMI1//SYN1//DENND2D//CASP3//DPH6//REPS2//RASAL2//DUSP18//SLC25A53//ACPP//ARL8B//GRIK5//LRRTM1//ELOVL6//ZDHHC11//FMO1//CYP26B1//TRAM2//SCYL3//HS3ST1//CLEC4C//RTKN2//MYOT//MSR1//PLEKHB2//PLS1//DDIT4L//ZNF425//FAP//GLRB//GMPPA//DND1//YY1AP1//CAMK1D//PLXDC1//AIF1L//GINS4//ABLIM2//TNFRSF8//NFE2L3// |
| GO:0005938 | cell cortex | Cellular component | 5 | 296 | 103 | 18698 | 3.0664523747048 | 0.0239531841577047 | 1 | 1.62063674654157 | 0.0485436893203883 | RTKN2//WIPF2//PLEKHH2//PLS1//NDFIP1// |
| GO:0043226 | organelle | Cellular component | 84 | 13597 | 103 | 18698 | 1.12148667860058 | 0.0249155031442945 | 1 | 1.60353033825073 | 0.815533980582524 | SMAP1//BMP2//SLC14A1//DYNC1I1//F5//GRIK5//SYN1//BRI3BP//GLS2//XAF1//MAPK3//TFAP4//AGMAT//ARL4C//ZNF267//DMRT2//CREBL2//DUSP18//ZNF425//MIER3//DLX3//DUSP9//DND1//NFATC2//NIP7//MIS18A//ACPP//YY1AP1//MAP2K7//CAMK1D//PSMD13//RORC//ZSCAN31//BMI1//ZFP36L1//TEAD3//ZMAT4//CASP3//EIF1AD//NFKBID//PRPF18//DPH6//ZNF697//ZNF845//TNFRSF8//NFE2L3//LRRTM1//TMEM214//ELOVL6//ZDHHC11//TRAM2//SCYL3//NDFIP1//IGLL5//CFTR//GMPPA//PLS1//ARL8B//AIF1L//SLC22A6//LTN1//NOL4//MKS1//WIPF2//DCX//ABLIM2//MYOT//PI4KB//PLEKHH2//SKIL//CLIP3//CLEC4C//MSR1//RTKN2//FMO1//CYP26B1//PLEKHB2//ARFIP1//GAL3ST1//GINS4//ADAT2//DENND2D//SLC25A53//HS3ST1// |
| GO:0043231 | intracellular membrane-bounded organelle | Cellular component | 70 | 10864 | 103 | 18698 | 1.16967770993895 | 0.0254036667784403 | 1 | 1.59510359256474 | 0.679611650485437 | BRI3BP//GLS2//XAF1//MAPK3//TFAP4//AGMAT//ARL4C//ZNF267//DMRT2//CREBL2//DUSP18//ZNF425//MIER3//DLX3//DYNC1I1//DUSP9//GRIK5//DND1//NFATC2//NIP7//MIS18A//ACPP//YY1AP1//MAP2K7//CAMK1D//PSMD13//RORC//ZSCAN31//BMI1//ZFP36L1//TEAD3//ZMAT4//CASP3//EIF1AD//NFKBID//PRPF18//DPH6//ZNF697//ZNF845//TNFRSF8//NFE2L3//LRRTM1//TMEM214//ELOVL6//ZDHHC11//SCYL3//SYN1//NDFIP1//TRAM2//CFTR//ARL8B//PI4KB//F5//SLC25A53//GINS4//PLEKHH2//ADAT2//WIPF2//SKIL//DENND2D//SLC14A1//NOL4//FMO1//CYP26B1//ARFIP1//GAL3ST1//CLIP3//HS3ST1//SMAP1//BMP2// |
| GO:0097060 | synaptic membrane | Cellular component | 5 | 317 | 103 | 18698 | 2.86331199656978 | 0.0309370394321134 | 1 | 1.50952124913102 | 0.0485436893203883 | GRIK5//CHRNA1//GLRB//LRRTM1//SHC4// |
| GO:0034707 | chloride channel complex | Cellular component | 2 | 50 | 103 | 18698 | 7.26135922330097 | 0.0310122982002628 | 1 | 1.50846604870272 | 0.0194174757281553 | GLRB//CFTR// |
| GO:0098590 | plasma membrane region | Cellular component | 11 | 1069 | 103 | 18698 | 1.86798296202785 | 0.0332513868972514 | 1 | 1.47819023579661 | 0.106796116504854 | SLC14A1//SLC22A6//CFTR//FAP//AIF1L//MAPK3//GRIK5//CHRNA1//GLRB//LRRTM1//SHC4// |
| GO:0098794 | postsynapse | Cellular component | 6 | 450 | 103 | 18698 | 2.42045307443366 | 0.0381809531626944 | 1 | 1.41815323392738 | 0.058252427184466 | CHRNA1//GLRB//GRIK5//LRRTM1//SHC4//SYN1// |
| GO:0120025 | plasma membrane bounded cell projection | Cellular component | 17 | 1980 | 103 | 18698 | 1.55862508580955 | 0.0424368917841779 | 1 | 1.37225643274463 | 0.16504854368932 | CHRNA1//DCX//GLRB//TNFRSF8//PLEKHH2//FAP//SCYL3//MAPK3//LRRTM1//GRIK5//PLXDC1//SYN1//NDFIP1//ARL4C//ACPP//AIF1L//MKS1// |
| GO:0043227 | membrane-bounded organelle | Cellular component | 78 | 12616 | 103 | 18698 | 1.12235656986659 | 0.0431744313314867 | 1 | 1.3647733739973 | 0.757281553398058 | SMAP1//BMP2//SLC14A1//FMO1//CYP26B1//DYNC1I1//BRI3BP//GLS2//XAF1//MAPK3//TFAP4//AGMAT//ARL4C//ZNF267//DMRT2//CREBL2//DUSP18//ZNF425//MIER3//DLX3//DUSP9//GRIK5//DND1//NFATC2//NIP7//MIS18A//ACPP//YY1AP1//MAP2K7//CAMK1D//PSMD13//RORC//ZSCAN31//BMI1//ZFP36L1//TEAD3//ZMAT4//CASP3//EIF1AD//NFKBID//PRPF18//DPH6//ZNF697//ZNF845//TNFRSF8//NFE2L3//LRRTM1//TMEM214//ELOVL6//ZDHHC11//SCYL3//SYN1//NDFIP1//F5//TRAM2//IGLL5//CFTR//GMPPA//PLS1//ARL8B//AIF1L//SLC22A6//PI4KB//SKIL//CLIP3//CLEC4C//MSR1//PLEKHB2//ARFIP1//GAL3ST1//SLC25A53//GINS4//PLEKHH2//ADAT2//WIPF2//DENND2D//NOL4//HS3ST1// |
